# Downregulation of ICCs and PDGFRα+ cells on colonic dysmotility in hirschsprung disease

**DOI:** 10.3389/fped.2022.975799

**Published:** 2023-01-09

**Authors:** Aiming Gu, Zhihao Wu, Peng Wang, Jun Liu, Jianfeng Wang, Qianqian Wang, Jie Chen

**Affiliations:** ^1^ Department of Neurology, Affiliated Hospital of Jiaxing University, Jiaxing, China; ^2^ Department of Pediatric Surgery, Xin Hua Hospital, Affiliated to Shanghai Jiao Tong University School of Medicine, Shanghai, China; ^3^ Department of Pediatric Surgery, Jiaxing Maternity and Child Health Care Hospital, Affiliated to Jiaxing College, Jiaxing, China; ^4^ Department of Pediatric Surgery, Shanghai Children's Medical Center, Shanghai Jiao Tong School of Medicine, Shanghai, China

**Keywords:** hirschsprung disease, colonic dysmotility, interstitial cells of cajal, platelet-derived growth factor receptor-α positive cells, smooth muscles

## Abstract

**Background:**

To investigate the effect of the distribution and expression of interstitial cells of Cajal (ICCs) and platelet-derived growth factor receptor-α positive (PDGFRα+) cells in different colon segments on colonic motility in children with Hirschsprung disease (HSCR).

**Methods:**

Smooth muscles of the narrow and dilated segments of the colon were obtained from 16 pediatric patients with HSCR. The proximal margin was set as the control section. The mRNA and protein expressions of c-Kit, PDGFRα, ANO1, and SK3 channels were examined. Circular smooth muscle strips of the colon were prepared for performing electrophysiology experiments using electric field stimulation (EFS) and intervention from different drugs (TTX, NPPB, Apamin, L-NAME, and CyPPA).

**Results:**

The mRNA and protein expressions of c-Kit, ANO1, PDGFRα, and SK3 were much lower in the narrow segment than those in the dilated and proximal segments of the colon. The narrow segment showed a considerably spontaneous contraction of the muscle strip. After the EFS, the relaxation response decreased from the proximal to the narrow segment, whereas the contraction response increased. TTX blocking did not cause any significant changes in the narrow segment. In contrast, when NPPB, Apamin, L-NAME, and CyPPA were used to intervene in the muscle strips, the proximal segment showed a more sensitive inhibitory or excitatory response than the narrow segment.

**Conclusions:**

Downregulation of the ICCs and PDGFRα+ cells from the proximal to narrow segment may be responsible for the dysmotility of the colon in pediatric HSCR.

## Introduction

Hirschsprung disease (HSCR) is a gut motility disorder characterized by congenital aganglionosis that affects the distal bowel (1). The incidence rate of this life-threatening disease is approximately 1/5,000 in neonates, presenting with delaying the passage of meconium, intractable constipation, and abdominal distension (2, 3). The primary treatment for HSCR involves a radical pull-through operation (4). However, recent studies have reported several postoperative colonic dysmotility complications such as constipation, soiling, and recurrent enterocolitis at long-term follow-ups (5–9). However, the mechanism of colonic dysmotility is still not completely understood.

Normal gut motility depends on the coordinated interaction of the enteric nervous system (ENS) with smooth muscle cells (SMCs), interstitial cells of Cajal (ICCs), and platelet-derived growth factor receptor-α positive (PDGFRα+) cells, which constitute the SIP syncytium, regulate the alimentary tract by propagating electrical signals through its smooth muscle layers and gap junctions (10, 11). Puri et al. showed that the distribution of PDGFRα+ cells of the distal bowel decreased in HSCR (12, 13). In a previous study, we showed the downregulation of the c-Kit and calcium-activated chloride channel anoctamin 1 (ANO1) in ICCs and the upregulation of SK3 channels in PDGFRα+ cells using a partial colon obstruction (PCO) mouse model (14). However, the mechanism through which the ENS regulates SIP syncytium in colonic motility at different segments in pediatric HSCR has been rarely reported.

In this study, we investigate changes in the mRNA and protein expressions of ICCs/ANO1 and PDGFRα+ cells/SK3 in different segments of the colon in HSCR. Using the electrophysiological method, we hypothesize that the regulatory imbalance between the ENS and the SIP syncytium of the different colonic segments may be responsible for colon dysmotility.

## Materials and methods

### Specimen collection

The study conformed with the Declaration of Helsinki and was approved by the Ethics Committee of Xin Hua Hospital Affiliated with Shanghai Jiao Tong University School of Medicine (approval number: XHEC-D-2018-1052). Written informed consent was obtained from the parents and/or legal guardians of all study participants. The long-segment and total colonic aganglionosis as well as those accompanied with associated syndromes or other malformations were excluded from this study. Finally, 16 pediatric patients with the full extent of the resected colonic specimens were selected; their clinical data are shown in Table 1. The specimens were dissected into the narrow, dilated, and proximal segments. The proximal segment contained normal ganglion cells, as confirmed by hematoxylin-eosin staining, and was set as the control group. The specimens were stored at 0°C in the Krebs solution and transferred to the laboratory immediately before the experiments.

**Table 1:** Clinical data of pediatric patients with HSCR.

Date	Sex	Age	Total length (cm)	Surgical procedure
17.4.12	M	3m	25	Soave
17.5.16	M	3m	25	Soave
17.5.23	M	7m	30	Soave
17.5.3	M	3y	30	Soave
17.5.31	M	7m	18	Soave
17.7.17	M	8m	25	Soave
17.9.4	F	6m	25	Soave
17.9.13	M	2y	29	Soave
17.10.30	F	1y	38	Soave
17.11.2	F	1y	28	Soave
17.11.16	F	3m	20	Soave
17.12.12	M	2y	32	Soave
17.12.21	M	1y	25	Soave
18.1.12	M	1y	30	Soave
18.3.5	M	1y	32	Soave
18.4.2	F	1y	25	Soave

M, male; F, female; m, month; y, year.

### Protein extraction and Western blot analysis

The colonic smooth muscle tissues were prepared by mechanically removing the mucosa and submucosa layers. The muscle tissue fragments were ground and homogenized in an ice-cold radioimmunoprecipitation assay buffer (1:10; Beyotime Chemical Co., Jiangsu, China) containing 1% phenylmethylsulfonyl fluoride (PMSF; Beyotime Chemical Co., Jiangsu, China). The resulting soluble and insoluble fractions were separated by centrifugation at 4°C at 12,000 rpm over 15 min. According to the manufacturer's protocols, the concentration of the supernatant was determined by the bicinchoninic acid (BCA) protein assay method (Beyotime Chemical Co., Jiangsu, China) with a standard curve generated by using known concentrations of bovine serum albumin. The samples were denatured at 100°C for 5 min. Equal amounts of proteins (30 µl per lane) were separated by 10% or 8% sodium dodecyl sulfate-polyacrylamide gel electrophoresis (SDS-PAGE) and subsequently transferred to polyvinylidene difluoride (PVDF) membranes. After blocking with 5% non-fat milk in 0.1% Tris-buffered saline/Tween 20 (TBST) for 1 h, the PVDF membranes were incubated overnight with primary antibodies at 4°C. The primary antibodies used were anti-c-Kit (1: 500; ab178527; Abcam, United States), anti-TMEM16A (1 : 500; ab84915; Abcam, United States), anti-PDGFRα+ (1 : 1,000; 3174; Cell Signaling Technology, United States), and anti-SK3 (1 : 500; ab192515; Abcam, United States). Anti-GAPDH (1 : 500; ab181602; Abcam, United States) was used as the loading control for normalizing the expression of target proteins. The primary antibodies were removed by TBST washing, and the HRP-linked anti-mouse antibody (1 : 1,000; 7076; Cell Signaling Technology, United States) or anti-rabbit antibody (1 : 1,000; 7074; Cell Signaling Technology, United States) was then used as the secondary antibody in the PVDF membrane at room temperature for 2 h. Subsequently, the membrane was transferred to a chemiluminescence cassette for blot visualization. Image J software (open-access software available from http://imagej.nih.gov/ij/) was used for quantifying the digital densitometry of the band intensity.

### RNA isolation and quantitative reverse transcription-PCR (qRT-PCR)

The total RNA from the colonic smooth muscle layers was extracted from the narrow and dilated segments as well as from the proximal segment using the RNA simple Total RNA Kit (Tiangen, Beijing, China). First-strand cDNA was synthesized by denaturation at 8°C for 3 min and then by annealing at 44°C for 60 min. It was then extracted from RNA at 92°C using the PrimeScript RT Reagent Kit with gDNA Eraser (Takara, Dalian, China). The qRT-PCR was performed by using the SYBR Green I assay (Light Cycler 480 SYBR Green I Master Mix, Roche Diagnostics, Mannheim, Germany). The initial denaturation was performed at 95°C, after which 55 cycles of amplification were carried out for each primer. Each cycle included denaturation at 95°C for 7 s, annealing at 55°C for 10 s, and extension at 72°C for 15 s. The expression of the target genes relative to the endogenous control GAPDH was calculated using the ΔCT method. Gene-specific primers are listed in Table 2.

**Table 2:** Gene primer sequences.

Name	Forward primer sequence (5'–3')	Reverse primer sequence (5'–3')
*ANO1*	ACTACCACGAGGATGACAAGC	TCTCTGCACAGCACGTTCC
*c-Kit*	CGTTCTGCTCCTACTGCTTCG	CCCACGCGGACTATTAAGTCT
*PDGFRα+*	TTGAAGGCAGGCACATTTACA	GCGACAAGGTATAATGGCAGAAT
*SK3*	AAGCGGAGAAGCACGTTCATA	CTGGTGGATAGCTTGGAGGAA
*GAPDH*	CTGGGCTACACTGAGCACC	AAG TGGTCGTTGAGGGCAATG

### Preparation of muscle strips and isometric force measurement

The colonic smooth muscle strips (approximately 8.0 mm × 2.0 mm × 2.0 mm) were cut along the circular axis. A silk thread was attached to both ends of the muscle strips. One end of the strips was suspended along the circular axis in 10-mL organ baths and immersed in warm (37°C) oxygenated (95% O2 and 5% CO2) Krebs solution, while the other end was fixed on a muscle tension transducer (JZJ01, range 30 g). The mechanical activity of the strips was recorded using an isometric force transducer (RM624°C, Chengdu, China) connected to an amplifier. To avoid the unnecessary clamp and pull of the colon tissue during the operation, the Krebs solution was replaced every 20 min. A tension of 3 mN was applied for equilibration for at least 1 h to restore the contractile activity after full relaxation. As a result, the strips displayed spontaneous contractions. The experiments included the following steps: (1) The electric field stimulation (EFS) was used to induce smooth muscle contraction, with the following parameters: constant voltage, 50 V; pulse width, 0.5 ms; continuous stimulation time, 20 s; and stimulation frequencies, 6, 9, and 12 Hz. (2) After the first electrical stimulation, the muscle strips were restored to the baseline, and TTX (a neuron blocker that acts as an exogenous inhibitor), NPPB (an ANO1 channel inhibitor), Apamin (an SK3 channel inhibitor), CyPPA (an SK3 agonist), and L-NAME [a nitric oxide (NO) synthase inhibitor] were added sequentially. Their strip response was recorded accordingly.

### Solutions and drugs

The Krebs solution comprised the following (mM): NaCl, 118.5; KCl, 4.5; MgCl2, 1.2; NaHCO3, 23.8; KH2PO4, 1.2; dextrose, 11.0; and CaCl2, 2.4. NPPB, Apamin, and L-NAME were purchased from Tocris Bioscience (Ellisville, MO, United States).

### Statistical analysis

The data are shown as the means ± standard deviation. Paired *t*-test and ANOVA analysis with the LSD method were applied using a standard statistical software package (SPSS 20.0). A *P*-value < 0.05 was considered to be statistically significant.

## Results

### Reduced mRNA and protein expression levels of c-kit/ANO1 and PDGFRα/Sk3 in HSCR

**Figure 1:**
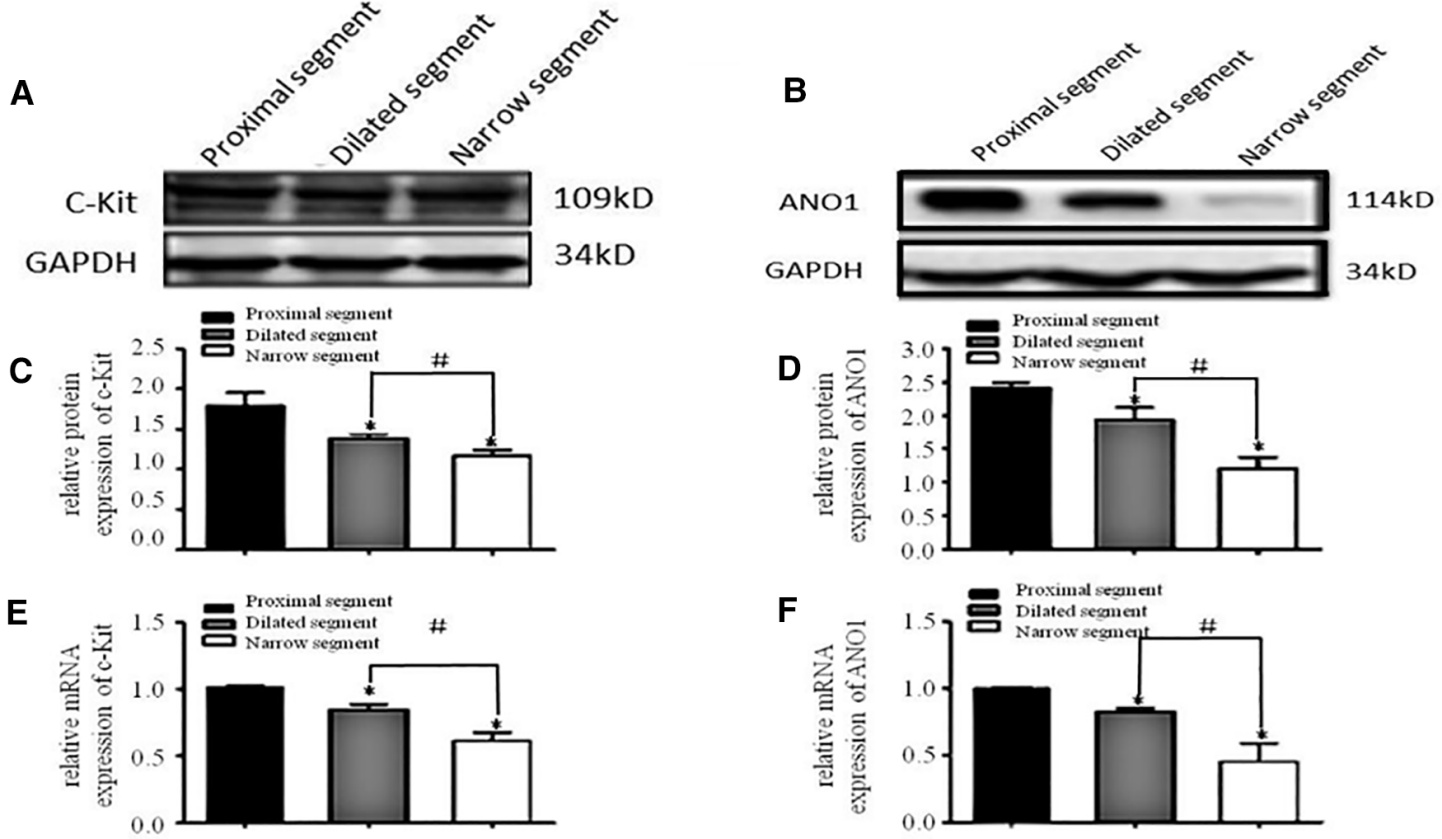
mRNA and protein expressions of c-Kit and ANO1. (**A,B**) Electrophoresis identification. (**C,D**) Significant difference in the relative expression of the proteins in all the three segments (narrow, dilated, and proximal) of the colon. (**E,F**): mRNA expressions of c-Kit and ANO1 in the three segments. *P < 0.05 vs. proximal segment; #*P* < 0.05 dilated vs. narrow segments.

**Figure 2:**
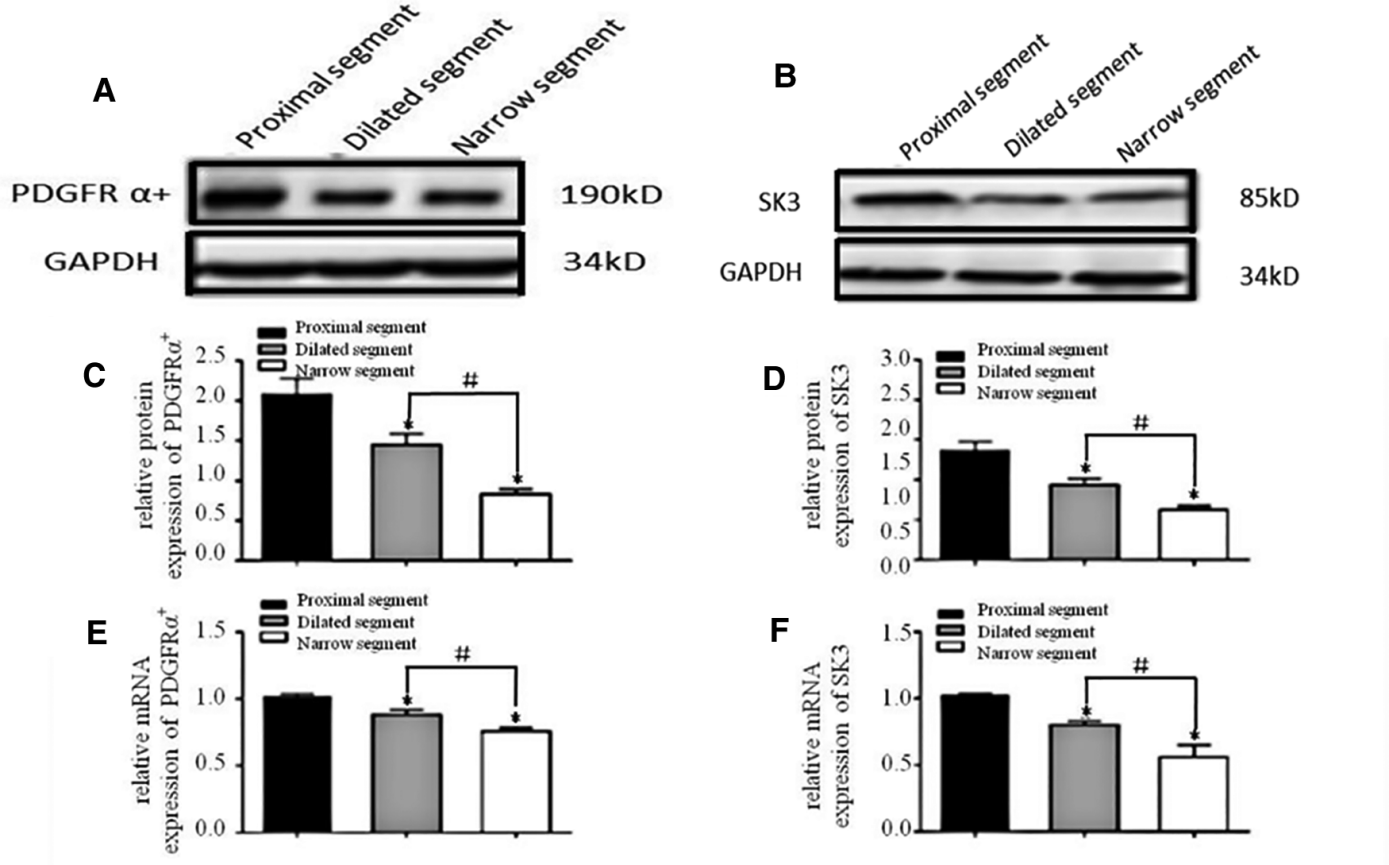
mRNA and protein expressions of PDGFRα and SK3. (**A,B**): Electrophoresis identification. (**C,D**): Significant difference among the relative expressions of proteins in the three segments (narrow, dilated, and proximal). (**E,F**): mRNA expressions of PDGFRα and SK3 in the three segments. *P < 0.05 vs. proximal segment; #*P* < 0.05 dilated vs. narrow segments.

A specific band at 110–115 kDa was identified for c-Kit (Figure 1A). The band for ANO1 was detected between 114 and 120 kDa (Figure 1B). PDGFRα was found with a band between 188 and 192 kDa (the predicted molecular weight was 190 kDa) (Figure 2A). SK3 had a band between 80 and 90 kDa (the predicted molecular weight was 85 kDa) (Figure 2B). The relative protein expressions of c-Kit/ANO1 and PDGFRα/SK3 in the narrow segment were significantly lower than those in the dilated and proximal segments (Figures 1C,D, 2C,D). The mRNA expression of c-Kit/ANO1 and PDGFRα/SK3 in the narrow segment was significantly lower than that in the dilated and proximal segments (Figures 1E,F, 2E,F).

### Abnormal contraction of colonic smooth muscle in HSCR with TTX and EFS

**Figure 3:**
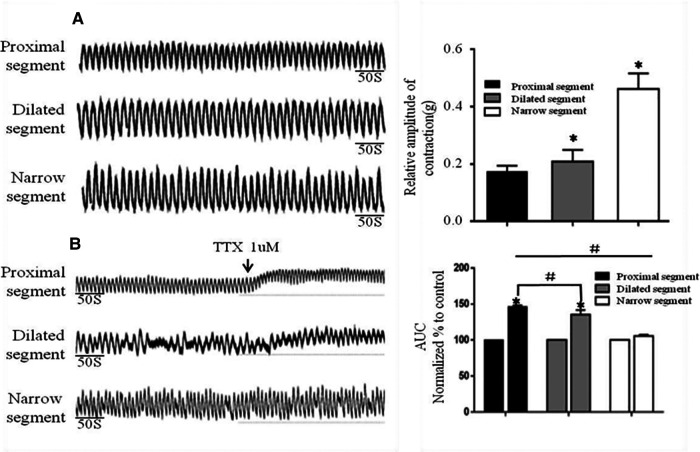
Spontaneous contractions of the smooth muscle of the colon without and with TTX blocker. (**A**): Significant differences among the spontaneous contractions in the narrow segment under static station with no intervention. *P < .05 vs proximal segment. (**B**): AUC alternation of each segment after the addition of the TTX blocker. *P < .05 vs before TTX; # *P* < .05 vs proximal segment.

**Figure 4:**
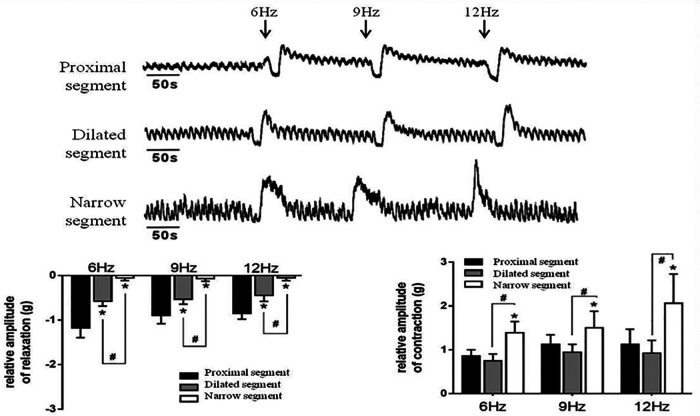
Effects of EFS-induced on smooth muscle contractions of the colon. Relaxation and subsequent contractile response after EFS in the proximal segment; relaxation response almost disappeared in the narrow segment. *P < 0.05 vs. proximal segment; #<0.05 dilated vs. narrow segments.

Strips of different colonic segments showed spontaneous contractions with TTX and EFS. The frequency and amplitude of the wave in both the narrow and dilated segments were greater than those in the proximal segment (Figure 3A). When TTX is added, the wave baseline of the proximal and dilated segments shifted upward and the area under the curve (AUC) of the proximal segment significantly increased compared to that of the dilated segment. In contrast, the narrow segment remained unaffected (Figure 3B). Under EFS, transient relaxations were present in both the proximal and dilated segments with a significant difference in the AUC. In contrast, the narrow segment did not show any relaxation, although it showed increased subsequent contraction compared to that shown by the proximal and dilated segments. However, no significant difference in the AUC was found between the latter two segments (Figure 4).

### Effect of NPPB, L-NAME, Apamin and CyPPA on the spontaneous contractions

**Figure 5:**
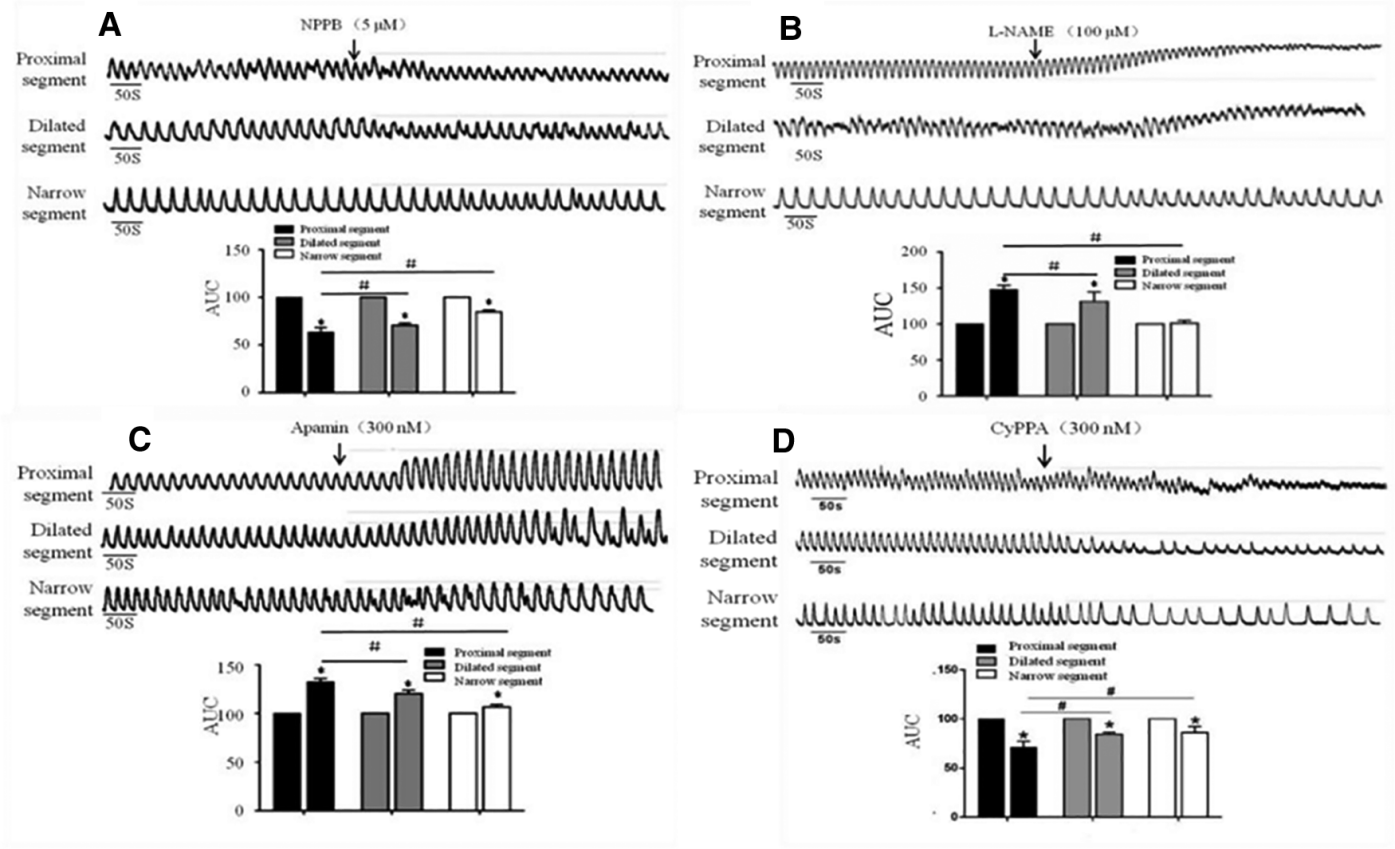
Effects of NPPB, L-NAME, apamin, and CyPPA on the smooth muscle contractions of the colon. A significant response was observed in the proximal segment compared to that in the narrow segment after the administration of (**A**) NPPB, (**B**) L-NAME, (**C**) Apamin, and (**D**) CyPPA, *<0.05 vs. before drugs; #<0.05 vs. proximal segment.

After the addition of NPPB (5 μmol/L), the AUC of the dilated and narrow segments significantly reduced in comparison to that of the proximal segment (Figure 5A). The proximal and dilated segments clearly showed increased AUC in response to L-NAME (100 μmol/L), while the narrow segment did not show any changes (Figure 5B). In contrast, all parts of the colon showed enhanced AUC after the addition of Apamin (300 nmol/L); the proximal segment showed significantly increased AUC than that of the dilated and narrow segments (Figure 5C). In contrast, administration of 300 nmol/L of CyPPA decreased the AUC of all the three segments, although the proximal segment showed a greater decrease than the dilated and narrow segments (Figure 5D).

## Discussions

The study investigated the effect of the downregulation of the ENS on the SIP syncytium in different segments of the colon of pediatric HSCR using the electrophysiological method, which may be responsible for the colonic dysmotility after the pull-through operation.

The pathological change caused by the absence of ganglion cells and the thickening of cholinergic nerve fibers under excessive acetylcholine secretion is responsible for HSCR (15, 16). The frequency and amplitude of the spontaneous contraction wave in the narrow segment greatly increased compared to that in the proximal segments. As the EFS can promote the release of both inhibitory and excitatory neurotransmitters (17), the proximal colon showed strong transient relaxation and contraction responses, demonstrating that the responses induced by the EFS were caused by the release of the inhibitory and excitatory neurotransmitters. In contrast, the narrow segment showed almost complete disappearance of the relaxation response and a much more intense contraction response than that of the proximal segment. We used TTX to block the conduction of exogenous neurotransmitters. The muscular tension in the proximal and dilated segments increased significantly after the TTX addition, whereas no change was observed in the narrow segment, indicating that the enteric nerves may be damaged, which is consistent with the neuropathology of HSCR. However, irrespective of the inhibition caused by the administration of TTX and/or EFS, the spontaneous contractions comprising rhythmic electric slow waves were always present in each colonic segment, implying that the SIP syncytium in the HSCR colon still plays a key role in regulating the contractile rhythm.

Koh et al. first proposed the concept of SIP syncytium, believing that the rhythmic contraction of gastrointestinal SMCs results from the collaboration among the external nerves, intestinal plexus, and syncytium (18). Recent studies have also investigated the role of ICCs and PDGFRα+ cells and dysmotility of the stenotic colon in the development of HSCR (12, 13, 19). In this study, we showed that the mRNA and protein expressions of c-Kit/PDGFR were downregulated from the proximal segment to the narrow one. Our result is in accord with that reported by O'Donnell et al. (13).

As the NO is a primary inhibitor of the ENS, its target cells are ICCs (20). We used L-NAME to block the synthesis of NOS. We observed significantly increased tension of spontaneous contraction in the proximal segments compared to that in the dilated segments; in contrast, the contraction in the narrow segment remained unchanged. This observation confirmed that neuronal NO synthase (nNOS) may also have a role to play in the relaxation response induced by the EFS. We presumed that two factors may be responsible for the response of the narrow segment: (i) the NO neurons in the narrow segment are either damaged or absent and cannot release NO; and (ii) the number of target cells of NOS in the narrow segment such as ICCs decreased.

ANO1 is a functional protein specifically expressed in ICCs—the key ion channel that produces the pacemaker current in the gastrointestinal tract responsible for its motility (21). Coyle et al. investigated that the protein expression of ANO1 reduced from the ganglionic colon section to the aganglionic colon (22). In this study, we also observed that the mRNA and protein expression of ANO1 significantly decreased from the proximal segment to the narrow one. As NPPB blocked the ANO1 channel, it significantly inhibited the spontaneous contraction of the colonic smooth muscle of all the three segments. The proximal segment is more sensitive to NPPB than the dilated and narrow segments, directly reflecting the damage of ICCs in the narrow segment.

The SK3 channel was highly expressed in PDGFRα+ cells, which play an important role in the relaxation of smooth muscle through gap junctions (23). In this study, both the mRNA and protein expressions of PDGFRα+ and SK3 in different segments of the colon were downregulated subsequently. These results are in good agreement with those of Coyle et al. (12, 13). Hence, we used Apamin, an SK3 channel blocker (24), and CyPPA, an SK3 channel agonist (25), to check the activity of PDGFRα+ cells in each segment. The results showed that Apamin enhanced the muscular contraction tension in all the three colonic segments, although the proximal segment was more sensitive to Apamin than the dilated and narrow segments. On the contrary, CyPPA greatly inhibited the contraction of the three segments, especially that of the proximal segment, indicating that the reduced activity of PDGFRα+ cells in the narrow segment.

In conclusions, this study showed that the abnormal distribution of ENS-ICCs/ANO1-SMC and ENS- PDGFRα+/SK3-SMC axes in different segments of the colon affected by HSCR have different characteristics. The number of ICCs and PDGFRα+ cells gradually decrease from the proximal segment to the narrow segment. Both c-Kit/ANO1 and PDGFRα/SK3 showed significantly reduced expressions in the narrow segment. The reduced inhibition of NO/ICCs from the proximal segment to the narrow one may be responsible for the dysmotility of the colon in pediatric HSCR.

## Data Availability

The original contributions presented in the study are included in the article/Supplementary Material, further inquiries can be directed to the corresponding author.
